# Prone Positioning and Molecular Biomarkers in COVID and Non-COVID ARDS: A Narrative Review

**DOI:** 10.3390/jcm13020317

**Published:** 2024-01-05

**Authors:** Savino Spadaro, Jose Daniel Jimenez-Santana, Riccardo La Rosa, Giorgia Spinazzola, Pilar Argente Navarro, Carlo Alberto Volta, Gaetano Scaramuzzo

**Affiliations:** 1 Department of Translational Medicine, University of Ferrara, 44124 Ferrara, Italy; riccardo.larosa@edu.unife.it (R.L.R.); vlc@unife.it (C.A.V.); scrgtn@unife.it (G.S.); 2 Anesthesia and Intensive Care Unit, Emergency Department, Azienda Ospedaliera Universitaria di Ferrara, 44124 Ferrara, Italy; 3 Department of Anaesthesiology, Hospital Universitari i Politécnic la Fe, 46026 Valencia, Spain; jimenez_josedanielsan@gva.es (J.D.J.-S.); argente_marna@gva.es (P.A.N.); 4 Department of Emergency, Anesthesiologic and Reanimation Sciences, Fondazione Policlinico Universitario Gemelli, IRCSS, 00168 Rome, Italy; giorgia.spinazzola@policlinicogemelli.it

**Keywords:** prone position, biomarkers, ARDS, COVID-19

## Abstract

Prone positioning (PP) represents a therapeutic intervention with the proven capacity of ameliorating gas exchanges and ventilatory mechanics indicated in acute respiratory distress syndrome (ARDS). When PP is selectively applied to moderate-severe cases of ARDS, it sensitively affects clinical outcomes, including mortality. After the COVID-19 outbreak, clinical application of PP peaked worldwide and was applied in 60% of treated cases, according to large reports. Research on this topic has revealed many physiological underpinnings of PP, focusing on regional ventilation redistribution and the reduction of parenchymal stress and strain. However, there is a lack of evidence on biomarkers behavior in different phases and phenotypes of ARDS. Patients response to PP are, to date, decided on PaO_2_/FiO_2_ ratio improvement, whereas scarce data exist on biomarker tracking during PP. The purpose of this review is to explore current evidence on the clinical relevance of biomarkers in the setting of moderate-severe ARDS of different etiologies (i.e., COVID and non-COVID-related ARDS). Moreover, this review focuses on how PP may modulate biomarkers and which biomarkers may have a role in outcome prediction in ARDS patients.

## 1. Background

Prone positioning (PP) represents a therapeutic strategy originally described in critically ill patients in 1976 by Margaret Piehl [1]. In almost fifty years, research has extensively focused on understanding the physiological effects of this maneuver. Prone positioning indeed mitigates the pathological alterations of acute respiratory distress syndrome (ARDS), affecting the outcome in moderate-to-severe cases. After initial reports of oxygenation improvement [2,3], cornerstone studies such as the PROSEVA trial demonstrated that PP could reduce mortality in ARDS [4].

Physiologically, the prone position takes advantage of the anatomical features of the chest wall and of the inhomogeneous ventro-dorsal distribution of lung disease, permitting the optimization of ventilation/perfusion distribution [5]. Additionally, hemodynamic changes, such as the recruitment of intrathoracic vasculature and better right ventricle function, may contribute to the overall benefits of PP [6].

To date, a consensus exists on considering a PaO_2_/FiO_2_ ratio < 150 mmHg with positive end-expiratory pressure (PEEP) > 5 cm H_2_O as a strong indication to initiate PP [7,8]. These conditions were recently included in the latest European guidelines on ARDS management [9]. Evidence also exists on the timing to start PP, pointing towards a greater benefit in the earlier initiation of the maneuver [10]. An earlier initiation of PP may lie in the optimization of ventilation to effectively overcome the regional pulmonary stress and strain before the occurrence of fibrotic alterations in the later stage of the disease [8,11,12]. Nevertheless, a delayed PP up to 14 days after starting invasive mechanical ventilation (IMV) would still provide some benefit, according to a recent retrospective analysis by Jackson et al. [13].

Despite the PROSEVA trial remaining the reference for PP interruption criteria (i.e., achieving PaO_2_/FiO_2_ > 150 mmHg with FiO_2_ < 0.6 and PEEP < 10 cm H_2_O in the supine position) [4], many aspects are still subject to debate. For example, the duration of a prone position to maximize its benefit is still controversial. Evidence supports that PP should be maintained for 12–16 h to obtain clinically relevant improvement in oxygenation and respiratory mechanics [14,15]. Karlis et al. [16] prospectively studied the differences between standard and prolonged PP in COVID-19 ARDS (C-ARDS) patients, finding no significant differences in respiratory mechanics improvement nor in 28-day mortality. In other studies, such as Okin et al. [17], prolonged PP in highly experienced teams led to lower mortality and a lower recurrence of PP. Indeed, an intermittent strategy for PP may lead to de-recruitment of lung parenchyma over time, although the need for repeating PP may be higher in severe ARDS [16]. Parker et al. recently explored the benefit of a single, prolonged round (i.e., more than 39 h) [18].

According to the most recent guidelines [9], cessation of the prone position is still guided by oxygenation improvement only. A recommendation exists on prolonging PP over 16 h of duration, when possible, to achieve the most benefit on clinical outcomes. However, prolonged PP also carries a higher risk for pressure injuries and damage to the nervous plexes and may also require deeper sedation and hamper enteral feeding [19]. It is possible that PaO_2_/FiO_2_ improvement does not fully reflect the changes in the lung when exposed to prolonged PP. Moreover, the mechanisms associated with the time dependency of the PP effect on mortality still represent an important research gap [15] and could be important for further understanding and advancement of PP management.

ARDS severity may progress to the point of being refractory to improvement with protective ventilation and a prone position. In these cases, extracorporeal support with veno-venous ECMO (vv-ECMO) may represent a life-saving therapeutic approach. Criteria for initiation of vv-ECMO in ARDS of different etiologies mirror the criteria designed for the EOLIA trial [19], which represents one of the cornerstone trials on this matter. Ultra-protective ventilation and minimization of lung injury could potentially mediate the effects of vv-ECMO on lung parenchyma [7,20]. Recent evidence shows no difference in survival outcomes of vv-ECMO patients; there is no difference between C-ARDS and ARDS of other pulmonary etiologies [21], and evidence lacks on the differential application of this strategy in different ARDS sub-phenotypes [9]. Recent trials have also explored the feasibility of the prone position in vv-ECMO patients, showing the potential safety of this combination and its positive impact on outcomes [22,23], although larger trials may be warranted on this matter.

The pandemic outbreak of COVID-19 represented an overwhelming burden for healthcare workers worldwide and challenged the approach to ARDS treatment, including the use of prone positioning. Before the pandemics, prone positioning was employed in up to 7.9% of ARDS cases (16.3% in severe ARDS) according to the LUNG SAFE study [24] and over 13.7% (32.9% in severe cases) in the APRONET study [25]. After the COVID-19 outbreak, clinical application of PP has reached 60% of treated cases, according to the PRoVENT-COVID study [26]. This outstanding increase has been linked to specific pathological changes pertaining to COVID-19 ARDS [27,28].

Biological markers have been extensively described for both COVID and non-COVID ARDS [29]. Biomarker expression patterns may reflect different stages and phenotypes of ARDS. These patterns may also reveal different responses to clinical interventions, including prone positioning.

The lack of a specific biomarker for ARDS is arguably one of the most important obstacles to progress in developing novel treatments. The purpose of this review is to describe current evidence on the association between different biomarker expressions and PP, highlighting peculiarities in COVID and non-COVID ARDS. This review also discusses the usefulness of biomarkers in prone positions to prevent complications associated with mechanical ventilation and as prognostic factors for weaning from MV, ICU length of stay, and mortality.

## 2. Oxygenation and Ventilatory Mechanics in Prone Position

**Figure 1:**
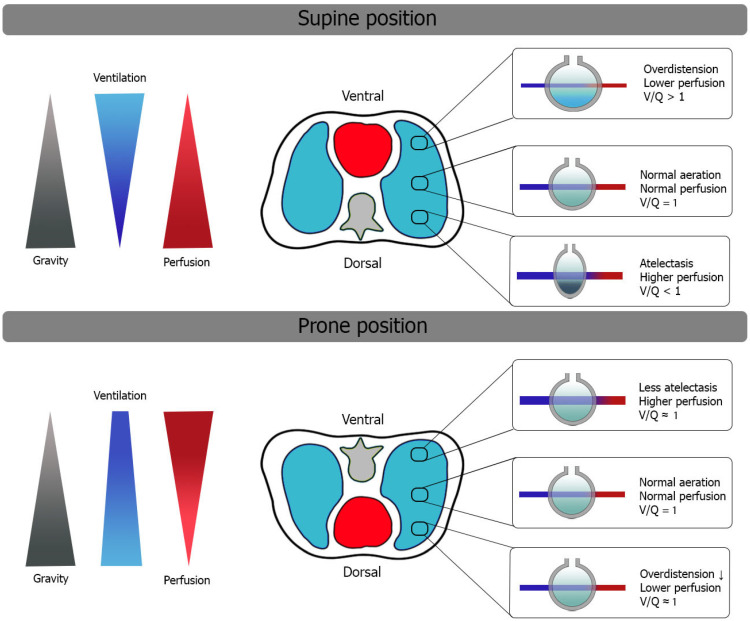
Changes in V/Q ratio after initiation of PP in mechanically ventilated patients with ARDS. Redistribution of ventilation more homogeneously helps the overall reduction of V/Q mismatch. See the text for details.

Improvement in gas exchange, as reported by the PaO_2_ increase, was the first therapeutic benefit described for the prone position [1]. The PaO_2_/FiO_2_ ratio continues to be a pivotal criterion in the starting prone position [9,30]. However, increased alveolar oxygen diffusion may be the result of several macro- and microscopic physiological modifications that the lung parenchyma undergoes during prone positioning [31]. Optimization of ventilatory/perfusion matching (V/Q ratio) and reduction of intrapulmonary shunt represent key elements of the process [32,33] (Figure 1).

The human rib cage consists of a ventral area composed of ribs and sternum and a dorsal area, including the spine and scapular surfaces, which is also less compliant with its intrinsic structure. Cranially, the thoracic cage is vertex-shaped, whereas caudally, it is limited by the diaphragm that separates the abdominal and thoracic cavities. Alveolar and vascular distributions are also inhomogeneous within the thorax; dependent regions contain higher alveolar and vascular densities with a limited contribution of gravity to the overall V/Q ratio [34,35,36,37].

In the supine position, gravity impacts the distribution of inflammatory edema, increasing its content in the dependent regions and redistributing ventilation preferentially to non-dependent areas, where transpulmonary pressure is lower and the ventral rib cage has greater compliance [30].

**Figure 2:**
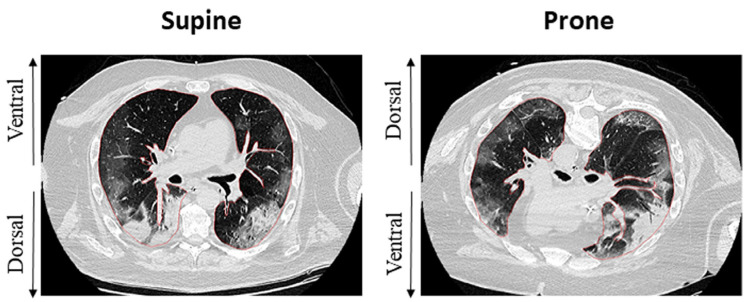
Effects of the prone position on recruitment and ventilation distribution in a patient with C-ARDS. Inhomogeneity of dorsal regions appears reduced after shift to PP, whereas ventral regions appear less recruited, possibly by means of reduced overdistention. Adapted from Fossali T. et al. [41].

These macroscopic modifications ultimately lead to dorsal collapse, which is evident on CT imaging of ARDS [38,39]. (Figure 2) Lung protective ventilation strategies with lower tidal volume and a moderate-to-high PEEP level demonstrated significant benefit in this setting [40]. However, in patients with moderate-severe ARDS, disease progression makes lung protective ventilation insufficient, requiring damaging pressures to maintain sufficient gas exchange, possibly meeting the criteria for starting in the prone position.

In the prone position, the inversion of the ventral and dorsal lungs redistributes the mechanical characteristics of the chest wall, decreasing its overall compliance [29] and changing its interaction with the lungs. Inversion of gravitational forces on parenchyma has been demonstrated on CT studies [30], and cardiac compression on lung parenchyma is also released. Changes in respiratory system compliance depend not only on the opening of previously unrecruited parenchyma but also on the improved mechanical behavior of already-opened alveoli [39,42]. When these changes occur together, the respiratory system moves towards a more favorable position, and total stress and strain may redistribute more homogeneously [36]. The reduction of airway plateau pressure after prone positioning thus acts as an indirect indicator of improved respiratory system compliance [42]. Pleural pressures also show a more homogeneous gravitational gradient in the prone position than in the supine position, possibly as a result of regional improvements in ventilation distribution [42,43]. Conversely, perfusion distribution does not change considerably in the prone position. The dorsal parenchyma still receives most of the blood flow even when turning to the non-dependent position, with a limited contribution from gravity [31,44].

Therefore, regional ventilation redistribution acts as the primary mechanism for V/Q homogeneization in the prone position. Santini et al. [45] demonstrated in an animal model that the prone position did not change the ventilated lung mass, i.e., no lung parenchyma was recruited. There was also no significant change in the overall elasticity of the respiratory system. However, there was an improvement in pulmonary elastance with the diversion of air from overinflated alveoli to poorly ventilated alveoli. Furthermore, this study showed not only the changes in the overall elastance of the respiratory system but also the measures of its components: the pulmonary elastance decreased and the elastance of the chest wall increased.

The redistribution of ventilation also plays an important role in the distribution of airway pressures. Therefore, in the prone position, the application of positive end-expiratory pressure (PEEP) has a lower probability of causing regional hyperinflation [46]. Protective ventilation is thus enhanced during prone positioning, providing better protection from ventilator-induced lung injury (VILI) [31,32,34]. Homogenization of ventilation may also reduce the alveolar hypoxemic reflex and may favor nitric oxide production in the capillaries of the posterior and inferior lung regions [35].

The prone position has demonstrated a strong impact on improving gas exchange in moderate-severe ARDS. Vollenberg et al. showed an improvement in P/F ratio > 15% after at least 9.5 h of PP [38]. However, this improvement in oxygenation apparently has no correlation with mortality. In early studies by Gattinoni et al. [2] and Guérin et al. [3], the simple improvement in oxygenation did not correspond to improved survival. Nevertheless, in C-ARDS, a response to prone positioning was associated with improved survival in several studies [47].

Hemodynamic changes also play an important role in the prone position [48]. Beneficial hemodynamic changes may indeed counteract some of the pathologic features of ARDS. Huang et al. [49] have reported the results of an echocardiographic assessment after an observational study of a large cohort of patients with COVID-19. They reflect the high incidence of pathologic echocardiographic findings in patients with moderate-severe C-ARDS. Huang et al. postulate that in C-ARDS, the heart suffers direct insult by the systemic inflammatory state (i.e., septic cardiomyopathy with LV systolic dysfunction), but also indirect damage caused by distress on the pulmonary vasculature (i.e., RV dysfunction, RV failure, and acute cor pulmonale (ACP)). In the post-hoc analysis of the ECHO-COVID study [50], they analyzed the different types of right ventricular involvement, concluding that of the wide variety of RV pathologies due to C-ARDS, ACP was associated with worse outcomes.

Jozwiak et al. [6] studied the hemodynamic effects of the prone position and found that if there is volume reserve, there is an improvement in the cardiac index due to an improvement in preload. They systematically found that there is a reduction in RV afterload. The factors that are related to the decrease in RV afterload are the recruitment of collapsed pulmonary microvasculature, lung recruitment, and the reduction of the hypoxic pulmonary vasoconstriction reflex due to better oxygenation and better distribution of regional ventilation.

Contradictory results have been reported regarding PaCO_2_ changes during PP. By homogenizing ventilation distribution, a reduction in alveolar dead space and arterial CO_2_ is expected. However, the reduction in rib cage compliance may lead to a lower Vt in the case of pressure-controlled ventilation, producing lower minute ventilation. On the other hand, in the case of volume-controlled ventilation, this reduction in rib cage compliance can lead to an increase in pleural pressure that hinders venous return and thus produces an increase in dead space due to reduced pulmonary regional perfusion [51]. The value of PaCO_2_ reduction as an indicator of net lung recruitment is such that it has been directly related to a decrease in 28-day mortality [38,40,52,53].

In conclusion, the prone position ameliorates gas exchange, ventilatory mechanics, and protective mechanical ventilation in moderate-severe ARDS. At the tissue level, this translates into less stress and strain on the lung parenchyma, potentially modulating inflammatory stimuli. Understanding these macroscopic changes in ventilatory mechanics helps understand the microscopic effects of PP, which may be reflected by the expression of different biomarkers.

## 3. Biomarkers in COVID ARDS and Non-COVID ARDS

ARDS is characterized by pathological alteration of the alveolar-capillary membrane (epithelial and endothelial damage) leading to pulmonary edema with protein-rich fluids (unfit for the alveolar and interstitial space), cell migration, and disseminated compressive atelectasis with lung collapse [54,55,56]. The inflammatory response that takes place in lung parenchyma arises from a primary insult and amplifies over time. Mechanical ventilation can potentially contribute to parenchymal stress and overall biotrauma [57]. Historically, several biomarkers in ARDS have been extensively analyzed in blood, bronchoalveolar lavage fluid (BALF), and exhaled breath [58,59].

The soluble receptor for advanced glycation end-products (sRAGE) represents an interesting example of a soluble plasma biomarker characterizing the acute inflammatory phase of ARDS [58]. This receptor is highly expressed by alveolar type-I cells and acts as an intercellular signaling molecule, mediating inflammatory response propagation. Increasing levels of sRAGE correlate with the severity of ARDS, potentially representing a marker of lung epithelial damage regardless of etiology [60,61]. More recently, sRAGE seems to correlate to the need for mechanical ventilation in C-ARDS [62].

Similarly to sRAGE, Surfactant Protein D (SP-D) behaves as a tissue-specific biomarker in ARDS, with an acute rise as epithelial damage occurs [63]. Interestingly, SP-D increases during acute lung injury and seems attenuated when protective mechanical ventilation is guaranteed, thus acting as indirect information on ventilator-induced lung injury (VILI) [64]. Moreover, expression of SP-D may hold more specificity to ARDS caused by viral and atypical pathogens than bacterial etiology [65]. However, the role of SP-D in determining the ARDS diagnosis remains controversial, as meta-analyses reveal [66,67].

Markers of the inflammatory cascade represent another valuable source of information for ARDS patients. Interleukin (IL) signaling plays a key role in inflammatory modulation. As a result, cytokines such as IL-8, IL-6, and IL-1B have been studied in different ARDS models [58]. IL-8 has been isolated in BALF of patients at risk for ARDS, and its levels correlate with disease severity [66,68]. IL-8 holds diagnostic potential for ARDS, especially when combined with other biomarkers such as SP-D and sRAGE [69]. Importantly, citokinic signaling involving IL-8, IL-6, IL-1B, and TNF-alpha is significantly modulated with protective lung ventilation, as was elegantly pointed out by Ranieri et al. [70]. Therefore, inflammatory overexpression may not only act as a marker of disease severity but also as an indirect source of information for parenchymal stress and strain.

A dysregulated inflammatory response is also a hallmark alteration in severe COVID-19 cases, especially when causing ARDS [71,72,73]. IL-6 elevation in plasma has been extensively studied in C-ARDS, showing a direct correlation with disease severity [71]. IL-6 also represents a key example of a biomarker becoming a pharmacological target [74]. Interestingly, IL-6 (i.e., Tocilizumab) therapeutic potential in non-COVID ARDS is still uncertain [75].

Systemic immune dysregulation has also been investigated as a potential therapeutic target for hemopurification (HP) strategies. Different HP techniques exist and can be tailored to patients’ needs [76,77]. The potential of this strategy lies in the direct modulation of the inflammatory cascade, which is a prominent feature of COVID-19-related ARDS (C-ARDS) [71]. Technological advancements have allowed a wider application of molecule absorption thanks to more biocompatible and efficient biomaterials, and the number of studies reporting more situations of clinical benefit is increasing, i.e., Hayanga et al. [78]. However, there is still insufficient evidence to support the application of HP in treatment guidelines, as larger studies addressing the main outcomes are needed to assess the biological and clinical efficacy of this therapy [79,80].

C-ARDS represents nowadays a distinct subphenotype of ARDS with specific pathogenic patterns. Indeed, SARS-CoV-2 has tropisms for molecules such as Angiotensin Converting Enzyme-2 (ACE2) and Transmembrane Serine Proteases (TMPRSS2) [81,82]. This mainly affects type-II alveolar cells, decreasing pulmonary surfactant production and hindering regeneration of the epithelium [72]. As alveolar damage progresses, clinical manifestations occur, followed by endothelial activation of small vessels with diffuse endotheliitis, widespread involvement of the microvasculature, and inflammatory cell infiltrates [83]. These alterations result in pulmonary vascular lesions and disseminated micro-thrombosis, significantly more frequent in C-ARDS than in classic ARDS [84,85].

Endothelial damage may, therefore, be a characteristic feature of C-ARDS. Indeed, the predominance of biomarker-measured endothelial damage over alveolar epithelial damage has been demonstrated with differential levels of biomarkers such as ANG-2 and ICAM-1 (intercellular soluble adhesion molecule-1) [72]. These biomarkers also have a correlation with clinical outcomes in mechanically ventilated C-ARDS patients [86].

At the molecular level, recent research has also focused on how SARS-CoV-2 may trigger an exaggerated inflammatory response and how this affects cellular functioning. Indeed, evidence points towards the possibility that SARS-CoV-2 may activate multiple pro-apoptotic signaling pathways, including lipid peroxidation and altered iron metabolism (i.e., ferroptosis) [87]. Radical oxygen species (ROS) are increasingly produced intracellularly in dysregulated inflammatory responses. When this happens, the production of altered cellular components such as malonyldialdehyde (MDA) may represent a biomarker of molecular damage [87]. Additionally, in vitro studies suggest that COVID-19 non-survivors show a higher propensity to oxydative stress on the endothelium [88]. These molecular pathways may trigger the production of free radicals and expose endotelial linings to cytokine cascades and altered immunization responses, finally creating the basis for the long-term damage often reported with COVID-19 [87,89].

Some biomarkers may also help to characterize further sub-phenotypes of ARDS. Indeed, hyper- and hypo-inflammatory sub-phenotypes have been described for classical ARDS based on the combination of multiple variables, including inflammatory cytokines [90]. The same holds true for C-ARDS, where differential expression of inflammatory cytokines may explain the inhomogeneous response to clinical treatments [91].

## 4. Prone Positioning and Biomarkers in COVID-19 and Non-COVID-19 ARDS

An ideal biomarker should give us relevant information about the onset and course of the pathology, should have a sensitivity and specificity close to 100%, and should provide information during the course of the disease on the response to treatment [92]. In ARDS, the goal is to find biomarkers that allow us to assess severity, the progression to more severe phenotypes, and predict outcomes (see Table 1 for a review of relevant biomarkers).

The fundamental characteristic of biomarkers is that they reflect the pathophysiology of the disease. In-depth knowledge of the molecular aspects of non-COVID ARDS and C-ARDS is what allows us to understand the role of biomarkers. In turn, the discovery of certain biomarkers in relation to each disease has helped in understanding and learning about these pathologies [92,93].

As mentioned, prone positioning is especially indicated in the diffuse alveolar damage phase of ARDS, when there is still lung parenchymal recruitability before fibrosis is reached. The prone position can allow the use of lower FiO_2_ due to better oxygenation, less pulmonary regional stress and strain, and better vascularization of the ventilated alveoli [85,94]. These improvements would presumably lead to a lower inflammatory state compared to the supine position, with less alveolar endothelial and epithelial damage.

Changes in ventilatory mechanics in the prone position are also found at a molecular level. Harmful mechanical ventilation induces the expression of molecular pathways in the lung, many of which are involved in the development of immunity, inflammation, apoptosis, cell communication, and the cytoskeleton [95,96]. Park et al. [97] evaluated molecular signaling pathways when rats with VILI were switched from the supine to the prone position. The authors evaluated differences between lung regions after prone positioning and found that prone positioning can dampen the molecular pathways that regulate the stress response and strain the lung parenchyma. Indeed, they found significant changes in the regional expression of MKP-1 and determined the functional effects of this by measuring the activity of the major MAPK inflammatory signaling pathway proteins.

Evaluating the effect of atelectasis on proteomics is fundamental to understanding the biological alterations generated by ARDS. Rashid et al. [98] studied proteomics following a model of acute lung damage generated with lipopolysaccharide (LPS) and endotoxin. They were able to reflect how atelectasis in these cases has a clear effect on increasing inflammation and dysfunction of the alveolar-capillary membrane. They detected a high expression of a wide range of pro-inflammatory interleukins (IL-6 and IL-20), signaling molecules (MAPK12 and STAT 1), inflammatory mediators (MPO, BTK, and RAGE), and chemokines (CXCL11 and CCL5), as well as increased leukocyte migration. As markers of alveolar-capillary dysfunction, they found extracellular matrix glycoproteins, vascular endothelial growth factor, and fibrinogen independently of the presence of systemic endotoxemia. Additionally, Endotoxemia promoted further metabolic changes in atelectasis with increased glycolytic enzymes, carbon metabolism, and glycolysis pathways [98].

Biomarkers could be an early warning tool in the management of patients with ARDS in a prone position. Its rapid availability may shed light on the evolution of complex ARDS cases and may help to monitor the response to the treatment.

Papazian et al. showed for the first time the difference in proinflammatory parameters in blood and BAL between the supine and prone positions. Their parameters studied were IL-1B, IL-6, IL-8, and TNF-a, as well as neutrophil levels. Papazian et al. found reduced levels of all these molecules except TNF-a, which would correspond to a lower level of epithelial damage and inflammation due to the beneficial effects of the prone position on recruitable lungs [99].

Chang et al. also investigated several biomarkers: IL-1b, IL-6, IL-10, and TNF-a. They studied 11 ARDS patients in the supine position and in the prone position. IL-1b was not measurable as it was in physiological ranges in all patients. No differences were found between the two groups in IL-10 or TNF-a values. IL-6 was the only blood parameter for which statistically significant differences were obtained, being lower in patients in the prone position and decreasing over time [100].

Musso et al. studied patients with C-ARDS under NIMV in supine and prone positions, differentiating according to mechanical power. In this case, patients undergoing prone position showed lower CRP, procalcitonin, and D-dimer levels at 7 days, which also decreased within each group as mechanical power decreased and gas exchange improved [101].

### Biomarkers in Prone Position in Relation to Main Outcomes

**Figure 3:**
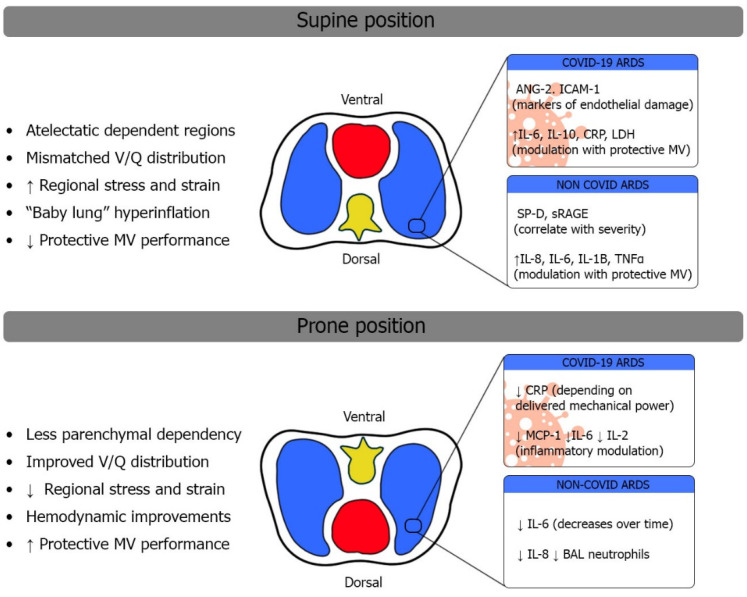
A schematic figure of clinically relevant biomarkers in supine and prone positions during ARDS. See the text for details.

Chang et al. [100] assessed the association of biomarkers with mortality. They established baseline, 24 h, and 72 h measurements. They assessed mortality at 14 and 28 days after the initiation of mechanical ventilation. In this study, IL-6 was shown to be a predictor of mortality at 14 days in both supine and prone patients, while it was not shown to be a predictor of mortality at 28 days. To this date, data are lacking regarding the predictive ability of biomarkers in the prone position in terms of the duration of mechanical ventilation or ICU stay (Figure 3).

## 5. Future Directions

Molecular biomarkers may represent the pathophysiological situation at any given time. Thus, a marker in the prone position should show a relationship with the patient’s response or non-response to this therapy. For example, a marker that tells us about epithelial damage due to cyclic overstretching in the supine position says that if the prone position achieves an improvement in ventilatory mechanics, this epithelial damage should decrease and be reflected in our parameter. The same is true with inflammatory or endothelial parameters.

The biomarkers that have proven useful in ARDS have a physiological correlation with the disease. Thus, the most studied blood markers include endothelial or epithelial damage molecules specific to the affected cells, cytokines, and other molecules related to the immune response to the inflammatory state. There is still little evidence on biomarkers in the prone position; so far, only IL-6 has been postulated in clinical studies as a useful biomarker. The peculiar inflammatory signature of COVID-19 may be addressed in future research and extend beyond IL-6, as it is possible that its unique molecular features (i.e., lipid metabolism changes and ferroptosis) may guide future treatments and prevent long-term sequelae.

With the current evidence, we can only take into account IL-6 as a useful marker for predicting short-term mortality in the prone position. Further studies are needed to define how the biomarkers can have a place in decision-making for patients in the prone position.

## 6. Conclusions

In conclusion, the prone position ameliorates gas exchange and ventilatory mechanics, enhancing protective mechanical ventilation in moderate-severe ARDS. At the tissue level, this translates into less stress and strain on the lung parenchyma, potentially modulating inflammatory stimuli. Biomarker expression patterns may reflect different stages and phenotypes of ARDS. These patterns may also reveal different responses to clinical interventions, including prone positioning. The evidence available to date only confirms IL-6 as a useful marker for predicting short-term mortality in the prone position.

Clinical studies of patients in the prone position may, in the future, test the predictive ability of biomarkers with proven evidence in ARDS. These may become a useful resource in clinical decision-making when this therapy is chosen in patients with moderate-severe ARDS.

## Figures and Tables

**Table 1:** Summary of articles in relation to biomarkers in the prone position in ARDS and selection of articles in relation to biomarkers in the supine position in ARDS. PP: Prone Position; BAL: Bronchoalveolar lavage; CRP: C reactive protein; SP-D: surfactant protein-D; RAGE: receptor for advanced glycation end-products; sRAGE: soluble receptor for advanced glycation end products; CC-16: club cell secretory protein; IL: Interleukin; TNF: Tumor necrosis factor; IGFBP7: Insulin-like growth factor binding protein 7; vWF: von Willebrand Factor; t-PA: tissue plasminogen activator; MMP: Matrix metalloproteinases; PCP III: procollagen peptide III; BNP: brain natriuretic peptide; ICAM-1: Intercellular Adhesion Molecule 1; GM-CSF: Granulocyte-macrophage colony stimulating factor; Ang-2: angiopoietin 2; VEGF: Vascular endothelial growth factor; MV: Mechanical Ventilation. CXCL10: C-X-C motif chemokine ligand 10; IP-10: Interferon gamma-induced protein 10.

**Biomarkers in Prone Position**				
NON-COVID ARDS	**Reference**	**Year**	**Biomarker**	**Main Outcome (s)**
	Papazian L	2005	Both in blood and in BAL: neutrophils, IL-8; IL-1B; IL-6; TNF-α.	To compare the physiologic (oxygenation) and proinflammatory effects of HFOV, prone positioning (PP), or their combination in severe ARDS
	Chan M-C	2007	IL-6, IL-10, IL-1B; TNF-α	To evaluate the safety of continuous PP ventilation and its effects on oxygenation and biomarkers. Compared with supine.
C-ARDS	Musso G	2023	CRP, Procalcitonin, D-Dimer	2º outcome: the effect of PP on Mechanical Power. Mechanical Ventilation (MV) parameters, biomarkers, days of MV, and mortality
	Lavillegrand J-R	2021	IL-1B, IL-6, CRP, IL-10, TNF-α, fibrinogen, limphocyte	To compare the immuno-inflammatory features according to organ failure severity and in-ICU mortality. 28D Mortality.
**Biomarkers in supine position**				
NON-COVID ARDS	Mrozek S.	2016	sRAGE	To characterize focal and non-focal patterns of lung CT-based imaging with biomarkers. ARDS phenotype, duration of MV, 28D, and 90D mortality
	Rosenberg CM	2023	Angiopoietin-2	To evaluate if plasma Ang-2 would be associated with the development of ARDS and poor clinical outcomes. Development of ARDS, severity of illness, and 30D mortality.
	Bendib I	2021	BAL fluid to serum ratio of IL8, BAL fluid to serum ratio of IL1, IL6, IP-10/CXCL, and IL10. TNFa, IFNg, ICAM-1, GM-CSF, VEGF, Angiopoetin1/2, RAGE, SP-D, HLA-DR CD8+ lymphocytes, and PD-1.	To evaluate the interrelation of ARDS/sepsis biomarkers in the alveolar and blood compartments and explore their association with clinical outcomes. Hospital mortality.
	Dong X	2020	Plasma IGFBP7, vWF, t-PA	To identify causal protein biomarkers for ARDS 28-D mortality using muti-stage Mendelian randomization.
	Headley AS	1997	TNF-α, IL1B, IL6, IL8,	To evaluate the relationships among clinical variables and biological markers of SIRS and patient outcome. Mortality.
	Yao-Ling-Lee	2021	IL6, IL8, IL1B, IL10, TNF-α, CRP	To determine whether biomarkers and endotoxins on the first day of ICU are associated with hospital mortality in severe pneumonia. Mortality
	Ware	2013	SP-D, RAGE, IL-8, CC-16, IL-6, ANG 2, MMP 1/3/9, PCPIII, BNP	To test if a biomarker panel would be useful for biologic confirmation of the clinical diagnosis of ARDS. The development of ARDS and the severity of the illness.
C-ARDS	Bain W	2021	IL-6, IL-8, IL-10	To compare key demographic and physiologic parameters, biomarkers, and clinical outcomes of COVID ARDS and non-COVID ARDS. 60D mortality, days of MV, MV parameters.
	Grasselli G	2020	D-dimer	To examine the functional and morphological features of COVID ARDS and to compare them with non-COVID ARDS. Mortality.
